# Reports to the UK Yellow Card Scheme 2000-2009 for medications used in paediatric rheumatology

**DOI:** 10.1186/1546-0096-9-S1-P237

**Published:** 2011-09-14

**Authors:** Daniel B Hawcutt, Clare Pain, Rachel Mulholland, Lisa M Cann, Gavin Cleary, Michael W Beresford, Eileen Baildam

**Affiliations:** 1 University of Liverpool, UK; 2 Alder Hey Children’s Hospital, Liverpool, UK

## Background & aims

The Medicines and Healthcare Regulatory Authority (MHRA) in the UK runs a national spontaneous reporting system (Yellow Card Scheme) to collect ‘suspected’ Adverse Drug Reactions (ADRs) for all medicines. To increase the data for paediatric medicines, current MHRA advice is to report all suspected ADRs in paediatric patients. Yellow Cards submitted between 2000 and 2009 were analysed for medications commonly used in paediatric rheumatology.

## Methods

Data on all UK spontaneous ‘suspected’ ADRs reported to the MHRA in patients <17 years from the years 2000-9 were supplied. Data included age, type of reaction, medication and type of reporter, but were unlinked. Data were only supplied on an individual drug if five or more suspected ADRs were reported in a calendar year. The cut-off values in the data supplied were in accordance with policy guidelines applied by the MHRA to preserve confidentiality of reporters and patients.

## Results

The number of reports of suspected ADRs to drugs commonly used in paediatric rheumatology per year are shown in Table 1. Adalimumab, tocilzumab & abatacept did not generate sufficient reports to feature in the dataset. There has been an increase in reporting over the 10 year period, with a median of 17 (2000-2) increasing to a median of 148 (2007-9). This increase is greater than the overall increase in reporting seen in the period (Hawcutt et al, submitted). However, the unlinked nature of the data supplied means that it is not possible to ascertain if the medications were prescribed in paediatric rheumatology patients.

**Table 1:** 

Drug	2000	2001	2002	2003	2004	2005	2006	2007	2008	2009	Total
ALEMTUZUMAB								9		22	**31**

AZATHIOPRINE					6				8		**14**

CYCLOPHOSPHAMIDE					11				18	36	**65**

CYCLOSPORIN	6			6		6	9	9	25	59	**120**

ETANERCEPT				8	8	22	22	20	14		**94**

ETOPOSIDE									5	7	**12**

INFLIXIMAB	5		7			10	5	10	11	20	**68**

METHOTREXATE	6		5	9	8	16	11	13	39	40	**147**

MYCOPHENOLIC ACID										8	**8**

PREDNISOLONE						6		12	14	19	**51**

RITUXIMAB									7	5	**12**

TACROLIMUS			8	12	7	10	13	12	7	18	**87**

## Conclusions

Medications commonly used in paediatric rheumatology generate suspected ADR reports in the UK. The overall reporting rate appears to be increasing, but reporting of biologics is lower than expected

